# Prospective Validation of a Rapid Host Gene Expression Test to Discriminate Bacterial From Viral Respiratory Infection

**DOI:** 10.1001/jamanetworkopen.2022.7299

**Published:** 2022-04-14

**Authors:** Emily R. Ko, Ricardo Henao, Katherine Frankey, Elizabeth A. Petzold, Pamela D. Isner, Anja K. Jaehne, Nakia Allen, Jayna Gardner-Gray, Gina Hurst, Jacqueline Pflaum-Carlson, Namita Jayaprakash, Emanuel P. Rivers, Henry Wang, Irma Ugalde, Siraj Amanullah, Laura Mercurio, Thomas H. Chun, Larissa May, Robert W. Hickey, Jacob E. Lazarus, Shauna H. Gunaratne, Daniel J. Pallin, Guruprasad Jambaulikar, David S. Huckins, Krow Ampofo, Ravi Jhaveri, Yunyun Jiang, Lauren Komarow, Scott R. Evans, Geoffrey S. Ginsburg, L. Gayani Tillekeratne, Micah T. McClain, Thomas W. Burke, Christopher W. Woods, Ephraim L. Tsalik

**Affiliations:** 1 Center for Applied Genomics and Precision Medicine, Duke University School of Medicine, Durham, North Carolina; 2 Hospital Medicine, Division of General Internal Medicine, Duke University School of Medicine, Durham, North Carolina; 3 Department of Biostatistics and Informatics, Duke University, Durham, North Carolina; 4 Duke Clinical Research Institute, Durham, North Carolina; 5 Department of Emergency Medicine, Henry Ford Hospital System, Detroit, Michigan; 6 Department of Pediatrics, Henry Ford Hospital System, Detroit, Michigan; 7 Department of Medicine, Henry Ford Hospital System, Detroit, Michigan; 8 Division of Pulmonary and Critical Care Medicine, Henry Ford Hospital System, Detroit, Michigan; 9 Department of Surgery, Henry Ford Hospital System, Detroit, Michigan; 10 McGovern Medical University of Texas Health, Houston; 11 Department of Emergency Medicine, The Ohio State University, Columbus; 12 Department of Emergency Medicine, Alpert Medical School of Brown University, Hasbro Children’s Hospital, Providence, Rhode Island; 13 Department of Pediatrics, Alpert Medical School of Brown University, Hasbro Children’s Hospital, Providence, Rhode Island; 14 Department of Emergency Medicine, University of California, Davis; 15 Division of Pediatric Emergency Medicine, UPMC Children’s Hospital of Pittsburgh, Pittsburgh, Pennsylvania; 16 Division of Infectious Diseases, Department of Medicine, Brigham and Women’s Hospital, Boston, Massachusetts; 17 Division of Infectious Diseases, Department of Medicine, Columbia University Irving Medical Center, New York, New York; 18 Department of Emergency Medicine, Brigham and Women’s Hospital, Boston, Massachusetts; 19 Department of Emergency Medicine, Newton-Wellesley Hospital, Boston, Massachusetts; 20 Department of Pediatrics, University of Utah, Salt Lake City; 21 Department of Pediatrics, University of North Carolina at Chapel Hill; 22 Department of Pediatrics, Northwestern University Feinberg School of Medicine, Chicago, Illinois; 23 The Biostatistics Center, George Washington University, Rockville, Maryland; 24 Division of Infectious Diseases, Department of Medicine, Duke University School of Medicine, Durham, North Carolina; 25 Medical Service, Durham Veterans Affairs Health Care System, Durham, North Carolina; 26 Emergency Medicine Service, Durham Veterans Affairs Health Care System, Durham, North Carolina

## Abstract

**Importance:**

Bacterial and viral causes of acute respiratory illness (ARI) are difficult to clinically distinguish, resulting in the inappropriate use of antibacterial therapy. The use of a host gene expression–based test that is able to discriminate bacterial from viral infection in less than 1 hour may improve care and antimicrobial stewardship.

**Objective:**

To validate the host response bacterial/viral (HR-B/V) test and assess its ability to accurately differentiate bacterial from viral infection among patients with ARI.

**Design, Setting, and Participants:**

This prospective multicenter diagnostic study enrolled 755 children and adults with febrile ARI of 7 or fewer days’ duration from 10 US emergency departments. Participants were enrolled from October 3, 2014, to September 1, 2019, followed by additional enrollment of patients with COVID-19 from March 20 to December 3, 2020. Clinical adjudication of enrolled participants identified 616 individuals as having bacterial or viral infection. The primary analysis cohort included 334 participants with high-confidence reference adjudications (based on adjudicator concordance and the presence of an identified pathogen confirmed by microbiological testing). A secondary analysis of the entire cohort of 616 participants included cases with low-confidence reference adjudications (based on adjudicator discordance or the absence of an identified pathogen in microbiological testing). Thirty-three participants with COVID-19 were included post hoc.

**Interventions:**

The HR-B/V test quantified the expression of 45 host messenger RNAs in approximately 45 minutes to derive a probability of bacterial infection.

**Main Outcomes and Measures:**

Performance characteristics for the HR-B/V test compared with clinical adjudication were reported as either bacterial or viral infection or categorized into 4 likelihood groups (viral very likely [probability score <0.19], viral likely [probability score of 0.19-0.40], bacterial likely [probability score of 0.41-0.73], and bacterial very likely [probability score >0.73]) and compared with procalcitonin measurement.

**Results:**

Among 755 enrolled participants, the median age was 26 years (IQR, 16-52 years); 360 participants (47.7%) were female, and 395 (52.3%) were male. A total of 13 participants (1.7%) were American Indian, 13 (1.7%) were Asian, 368 (48.7%) were Black, 131 (17.4%) were Hispanic, 3 (0.4%) were Native Hawaiian or Pacific Islander, 297 (39.3%) were White, and 60 (7.9%) were of unspecified race and/or ethnicity. In the primary analysis involving 334 participants, the HR-B/V test had sensitivity of 89.8% (95% CI, 77.8%-96.2%), specificity of 82.1% (95% CI, 77.4%-86.6%), and a negative predictive value (NPV) of 97.9% (95% CI, 95.3%-99.1%) for bacterial infection. In comparison, the sensitivity of procalcitonin measurement was 28.6% (95% CI, 16.2%-40.9%; *P* < .001), the specificity was 87.0% (95% CI, 82.7%-90.7%; *P* = .006), and the NPV was 87.6% (95% CI, 85.5%-89.5%; *P* < .001). When stratified into likelihood groups, the HR-B/V test had an NPV of 98.9% (95% CI, 96.1%-100%) for bacterial infection in the viral very likely group and a positive predictive value of 63.4% (95% CI, 47.2%-77.9%) for bacterial infection in the bacterial very likely group. The HR-B/V test correctly identified 30 of 33 participants (90.9%) with acute COVID-19 as having a viral infection.

**Conclusions and Relevance:**

In this study, the HR-B/V test accurately discriminated bacterial from viral infection among patients with febrile ARI and was superior to procalcitonin measurement. The findings suggest that an accurate point-of-need host response test with high NPV may offer an opportunity to improve antibiotic stewardship and patient outcomes.

## Key Points

**Question:**

What is the ability of a host gene expression test to accurately discriminate bacterial from viral infection among patients with acute respiratory illness?

**Findings:**

In this diagnostic study involving analysis of 616 children and adults with febrile acute respiratory illness of 7 or fewer days’ duration, the host response bacterial/viral test had up to 90% sensitivity, 82% specificity, and 98% negative predictive value for bacterial infection, which was significantly better than procalcitonin measurement.

**Meaning:**

The study’s findings suggest that an accurate point-of-need host response test with high negative predictive value may identify patients unlikely to have bacterial infection, offering a better antibiotic stewardship strategy than is currently available.

## Teaser

This diagnostic study assesses the ability of a host gene expression test to accurately discriminate between bacterial and viral infection among children and adults with acute febrile respiratory illness.

## Introduction

Acute respiratory illness (ARI) is the most common reason for urgent health care visits.^1,2^ Routinely available clinical information inadequately differentiates infections with bacterial causes from those with viral causes, contributing to high rates of inappropriate antibiotic medication use.^3,4,5,6,7^ Tests that reliably discriminate bacterial from viral infections could decrease diagnostic uncertainty, reduce inappropriate use of antibacterial therapy, and improve patient outcomes.

Pathogen identification tests, such as multiplexed syndromic panels, are important diagnostic tools but are unable to detect a bacterial or viral cause in most ARI cases.^8^ They also do not distinguish colonization from infection when a microbe is identified.^9,10^ Because the immunological responses to bacterial and viral infection are distinct, measuring the host response overcomes these limitations. Procalcitonin, the most widely used host biomarker, has exhibited mixed results for bacterial vs viral discrimination^11,12,13^ and for guiding the use of antibacterial therapy.^14,15^ Another approach involves measuring peripheral blood host gene expression, which can now be performed using clinically available platforms at the point of need.^16,17^

A previous study^18^ described the discovery of a gene expression signature discriminating bacterial from viral illness. This signature was further developed into a research-use-only host response bacterial/viral (HR-B/V) test via the widely used BioFire system and was evaluated using banked samples primarily from adults in a geographically limited environment.^16^ The present diagnostic study aimed to validate this HR-B/V test among a multisite diverse prospectively enrolled cohort and assess the superiority of the HR-B/V test over procalcitonin measurement. The study examined the feasibility and potential utility of a rapid point-of-need host response test to differentiate bacterial from viral respiratory infections.

## Methods

### Study Design

This study was approved by the institutional review boards of each participating hospital. All participants or legally authorized representatives provided written informed consent. The study followed the Standards for Reporting of Diagnostic Accuracy (STARD) reporting guideline for diagnostic studies.^19^

Participants were prospectively enrolled using convenience sampling from 10 US emergency departments: Duke University Hospital (Durham, North Carolina), Durham VA Health Care System (Durham, North Carolina), UNC Health Care (Chapel Hill, North Carolina), Henry Ford Health System (Detroit, Michigan), Brigham and Women’s Hospital (Boston, Massachusetts), University of California Hospital at Davis (Sacramento); Children’s Memorial Hermann (McGovern Medical, Houston, Texas), Hasbro Children’s Hospital (Providence, Rhode Island), University of Utah Medical Center (Salt Lake City), Children’s Hospital of Pittsburgh (Pittsburgh, Pennsylvania) and Newton-Wellesley Hospital (Newton, Massachusetts). Participants were enrolled from October 3, 2014, to September 1, 2019, followed by additional enrollment of patients with COVID-19 from March 20 to December 3, 2020. Included participants were 2 years or older with febrile ARI of 7 or fewer days’ duration. Acute respiratory illness was defined as having 2 or more qualifying symptoms or 1 qualifying symptom and at least 1 qualifying vital sign abnormality. A list of qualifying symptoms is available in eMethods in Supplement 1. Age was categorized as follows: children (2-11 years), adolescents (12-21 years), adults (22-64 years), and older adults (≥65 years). Patients with a known or suspected infection at any other anatomic site requiring antibacterial therapy were excluded. Charlson Comorbidity Index scores were calculated as previously reported.^20^

Enrollment ended before the onset of the COVID-19 pandemic. We therefore supplemented this analysis post hoc by analyzing blood RNA samples (PAXgene Blood RNA Tubes; QIAGEN) collected from 33 participants with acute SARS-CoV-2 infection through convenience sampling at Duke University or in a community setting from March to December 2020. All participants had positive results for SARS-CoV-2 on a polymerase chain reaction test, negative results for immunoglobin G, 7 or fewer days of symptoms, and no known bacterial coinfection.

### Reference Standard

**Figure 1.**
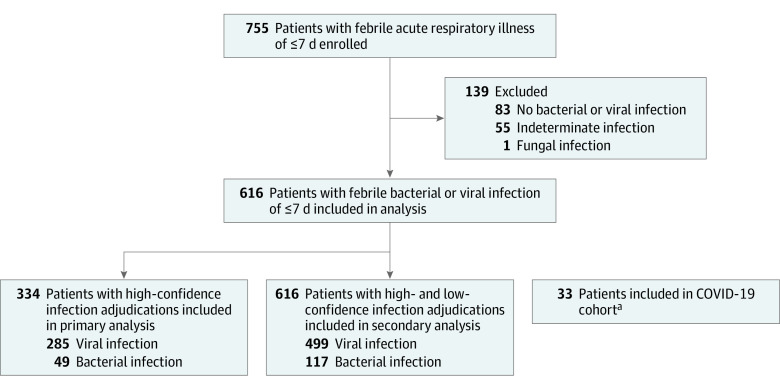
Study Flowchart ^a^The 33 patients in the COVID-19 cohort are an entirely separate group, enrolled independently of the rest of the cohort, which is why they are intentionally depicted in parallel to the other analysis groups.

Two adjudicators (including E.R.K., N.A., J.G., G.H., J.P., N.J., I.U., S.A., L. Mercurio, T.H.C., L. May, R.W.H., J.E.L., S.H.G., D.J.P., D.S.H., K.A., R.J., L.G.T., M.T.M., C.W.W., and E.L.T. along with other adjudicators who were not authors) from each enrollment site were randomly assigned to independently assess the likelihood of bacterial infection, viral infection, or no infection. Details regarding the adjudication process can be found in eMethods in Supplement 1. The primary analysis included participants with a high-confidence reference adjudication, which was defined as adjudicator concordance and the presence of an identified pathogen confirmed by microbiological testing (Figure 1; eTable 1 in Supplement 1). The secondary analysis involved all participants with a bacterial or viral infection, including those with a low-confidence adjudication, which was defined as adjudicator discordance or the absence of an identified pathogen in microbiological testing (Figure 1; eTable 1 in Supplement 1).

### Host Response Bacterial vs Viral Testing

The HR-B/V test detects 45 host messenger RNA targets using real-time quantitative reverse transcription polymerase chain reaction testing in approximately 45 minutes; the test was developed in collaboration with BioFire Diagnostics.^18^ RNA-preserved blood (PAXgene Blood RNA Tubes; QIAGEN) was processed according to manufacturer instructions and stored at −80 °C. The HR-B/V test was later performed by loading 100 μL of preserved blood (approximately 27 μL whole-blood volume) directly into research-use-only HR-B/V pouches (BioFire Diagnostics), which measured the relative abundance of target messenger RNAs normalized to the expression of 3 housekeeping genes (*DECR1*, *PPIB*, and *TRAP1*). Testing was conducted at Duke University. Details regarding the test and genes included in the panel have been previously published.^16^

### Statistical Analysis

Test measurements for the primary and secondary analysis included sensitivity, specificity, positive predictive value (PPV), negative predictive value (NPV), likelihood ratios, and area under the receiver operating characteristic curve.^21^ The HR-B/V test results were reported as probabilities of bacterial infection using a single threshold (probability score >0.41). We also grouped results into 4 likelihood categories: viral very likely (probability score <0.19), viral likely (probability score of 0.19-0.40), bacterial likely (probability score of 0.41-0.73), and bacterial very likely (probability score >0.73). The thresholds for these interpretive groups were derived from a previously characterized cohort.^16^

A procalcitonin level of 0.25 ng/mL or higher indicated bacterial infection. We also used previously described likelihood groups (<0.10 ng/mL indicated bacterial very unlikely, 0.10-0.24 ng/mL indicated bacterial unlikely, 0.25-0.50 ng/mL indicated bacterial likely, and >0.50 ng/mL indicated bacterial very likely).^22^ The median value was imputed for 25 participants with missing data.

We estimated the impact of age, sex, race and ethnicity, geographic area, hospital admission status, comorbidities, previous antibiotic medication use, illness duration, and illness severity for test performance using analysis of variance. Race and ethnicity were participant-defined or based on medical records when participants could not answer. Race was included as a covariate to determine whether it impacted the test, which measures immunological responses to infection. Bias-corrected and accelerated bootstrapping was used to estimate 95% CIs, and permutation tests were used to assess the statistical significance of the differences between performance measurements.^23^ All analyses were performed using Matlab software, version 2016b (Mathworks). Values for the area under the receiver operating characteristic curve were compared using the DeLong method.^24^ The significance threshold was 2-tailed *P* = .05.

## Results

### Participant Characteristics

Among 755 participants enrolled in the study, the median age was 26 years (IQR, 16-52 years); 360 participants (47.7%) were female, and 395 (52.3%) were male. Participants were racially and ethnically diverse, with 13 individuals (1.7%) identifying as American Indian, 13 (1.7%) as Asian, 368 (48.7%) as Black, 131 (17.4%) as Hispanic, 3 (0.4%) as Native Hawaiian or Pacific Islander, 297 (39.3%) as White, and 60 (7.9%) as unspecified (Table 1). Participants without an adjudicated bacterial or viral infection were excluded (Figure 1), resulting in 616 participants (median age, 24 years [IQR, 14-49 years]; 292 [47.4%] female and 324 [52.6%] male) included in the analyses. The median procalcitonin level among those participants was 0.05 ng/mL (IQR, 0-0.13 ng/mL) in patients with viral infection and 0.12 ng/mL (IQR, 0-0.47 ng/mL) in patients with bacterial infection (*P* = .004).

**Table 1.** Participant Demographic and Clinical Characteristics

Characteristic	Participants, No. (%)					
	Enrolled	Included in analyses	Primary analysis		Secondary analysis	
			Bacterial infection	Viral infection	Bacterial infection	Viral infection
Total, No.	755	616	49	285	117	499
Demographic						
Age, median (IQR), y	26 (16-52)	24 (14-49)	24 (16-36)	23 (10-48)	29 (19-53)	24 (12-48)
Sex						
Female	360 (47.7)	292 (47.4)	24 (49.0)	139 (48.8)	55 (47.0)	237 (47.5)
Male	395 (52.3)	324 (52.6)	25 (51.0)	146 (51.2)	62 (53.0)	262 (52.5)
Race and ethnicity						
American Indian	13 (1.7)	11 (1.8)	0	5 (1.8)	2 (1.7)	9 (1.8)
Asian	13 (1.7)	11 (1.8)	0	5 (1.8)	1 (0.9)	10 (2.0)
Black	368 (48.7)	293 (47.6)	23 (46.9)	135 (47.4)	46 (39.3)	247 (49.5)
Hispanic	131 (17.4)	110 (17.9)	8 (16.3)	58 (20.4)	19 (16.2)	91 (18.2)
Native Hawaiian or Pacific Islander	3 (0.4)	2 (0.3)	0	2 (0.7)	0	2 (0.4)
White	297 (39.3)	245 (39.8)	20 (40.8)	115 (40.4)	56 (47.9)	189 (37.9)
Unspecified	60 (7.9)	53 (8.6)	6 (12.2)	28 (9.8)	12 (10.3)	41 (8.2)
Clinical						
Symptom duration, median (IQR), d	3 (2-4)	3 (2-4)	3 (2-4)	3 (2-4)	3 (2-4)	3 (2-4)
Procalcitonin level, median (IQR), ng/mL	0.13 (0.08-0.32)	0.07 (0-0.16)	0.12 (0-0.47)	0.06 (0-0.15)	0.12 (0-0.47)	0.05 (0-0.13)
Charlson Comorbidity Index score, mean (SD)	1.37 (2.11)	1.19 (1.94)	1.35 (2.65)	1.04 (1.60)	1.74 (2.72)	1.06 (1.68)
ICU admission	35 (4.6)	23 (3.7)	2 (4.1)	12 (4.2)	5 (4.3)	18 (3.6)
Deaths	7 (0.9)	4 (0.6)	1 (2.0)	0	4 (3.4)	0
Receipt of antibacterial therapy before enrollment	103 (13.6)	87 (14.1)	17 (34.7)	26 (9.1)	43 (36.8)	44 (8.8)

Abbreviation: ICU, intensive care unit.

### Host Response Bacterial/Viral Test Performance

#### Primary Analysis

**Figure 2.**
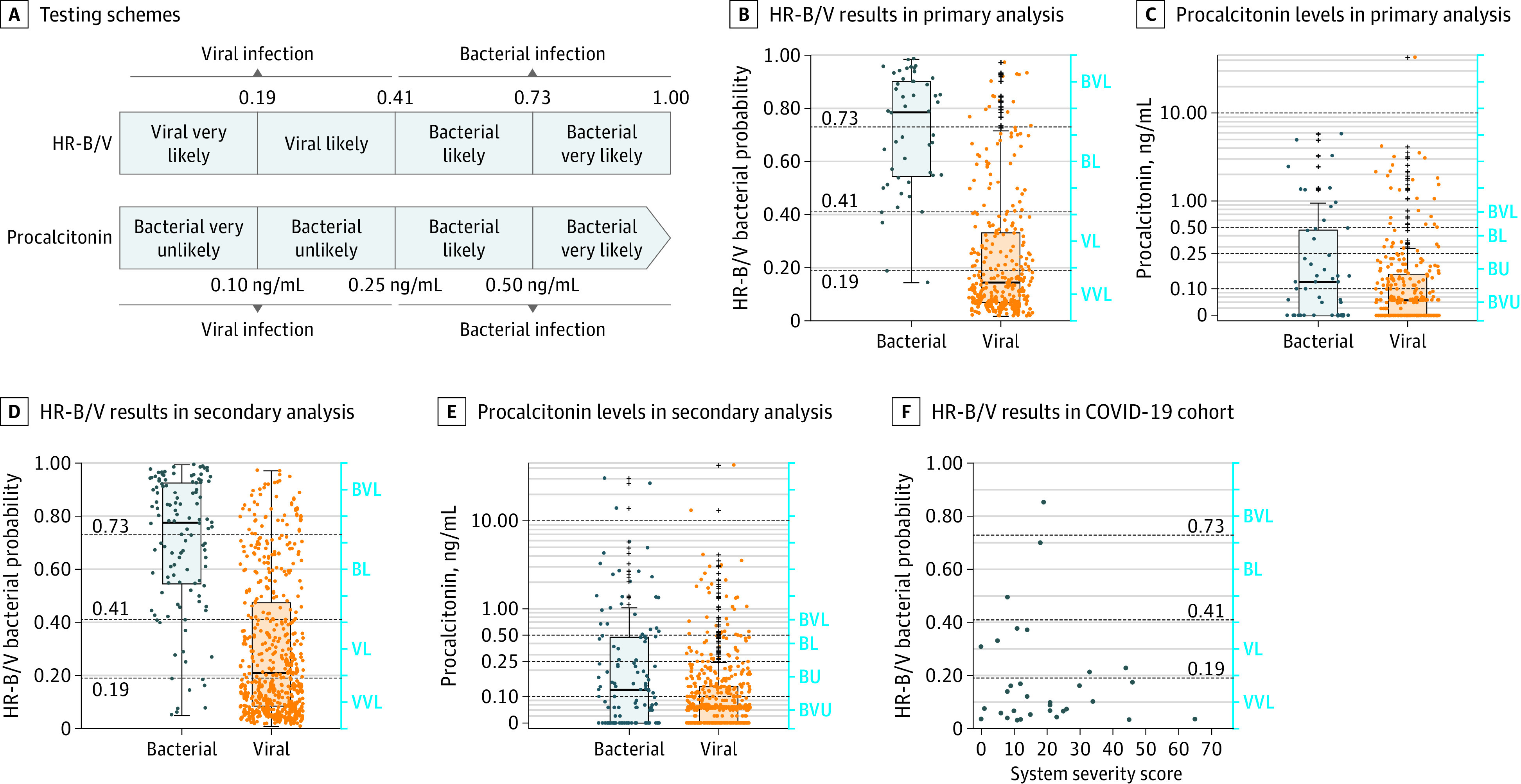
Test Performance A, Four-group likelihood schemes for the host response bacterial/viral (HR-B/V) test and procalcitonin measurement. In both cases (HR-B/V test vs procalcitonin measurement), results can also be interpreted in a dichotomous manner using a single threshold. B, The primary analysis cohort included 334 participants with high-confidence adjudications. The line within the box indicates the median value, the box indicates the first to third IQRs, the whiskers indicate the IQRs multiplied by 1.5, and the plus signs indicate outliers. C, The primary analysis cohort included 334 participants with high-confidence adjudications. The line within the box indicates the median value, the box indicates the first to third IQRs, the whiskers indicate the IQRs multiplied by 1.5, and the plus signs indicate outliers. D, The secondary analysis cohort included 616 participants with high- and low-confidence adjudications. The line within the box indicates the median value, the box indicates the first to third IQRs, the whiskers indicate the IQRs multiplied by 1.5, and the plus signs represent outliers. E, The secondary analysis cohort included 616 participants with high- and low-confidence adjudications. The line within the box indicates the median value, the box indicates the first to third IQRs, the whiskers indicate the IQRs multiplied by 1.5, and the plus signs indicate outliers. F, The COVID-19 cohort included 33 participants with acute SARS-CoV-2 infection. The dots in panels B to F indicate individual participants. BL indicates bacterial likely; BU, bacterial unlikely; BVL, bacterial very likely; BVU, bacterial very unlikely; VL, viral likely; and VVL, viral very likely.

The primary analysis included 334 participants (285 with viral infection and 49 with bacterial infection) with a high-confidence adjudication (eTable 1 in Supplement 1). Participants were first analyzed using a single threshold (probability score ≥0.41, indicating bacterial infection) (Figure 2A). In the primary analysis cohort, the HR-B/V test had sensitivity of 89.8% (95% CI, 77.8%-96.2%), specificity of 82.1% (95% CI, 77.4%-86.6%), and an NPV of 97.9% (95% CI, 95.3%-99.1%) for bacterial infection (Table 2; Figure 2B). Using a procalcitonin threshold of 0.25 ng/mL for bacterial infection, sensitivity was lower at 28.6% (95% CI, 16.2%-40.9%; *P* < .001) but specificity was higher at 87.0% (95% CI, 82.7%-90.7%; *P* = .006) compared with the HR-B/V test (Table 2; Figure 2C); the NPV was 87.6% (95% CI, 85.5%-89.5%; *P* < .001). The procalcitonin area under the curve was 0.59 (95% CI, 0.49-0.68) compared with 0.92 (95% CI, 0.87-0.94) for the HR-B/V test (*P* < .001) (eFigure in Supplement 1).

**Table 2.** Performance Characteristics of HR-B/V Test vs Procalcitonin Measurement for the Diagnosis of Bacterial Infection Using a Single Threshold

Analysis	% (95% CI)				LR^+^ (95% CI)	LR^−^ (95% CI)	AUROC (95% CI)
	Sensitivity	Specificity	PPV	NPV			
Primary analysis							
HR-B/V test	89.8 (77.8-96.2)	82.1 (77.4-86.6)	46.3 (39.8-53.0)	97.9 (95.3-99.1)	5.02 (3.85-6.55)	0.12 (0.05-0.29)	0.92 (0.87-0.94)
Procalcitonin measurement	28.6 (16.2-40.9)	87.0 (82.7-90.7)	27.5 (18.1-39.3)	87.6 (85.5-89.5)	2.20 (1.29-3.76)	0.82 (0.68-0.99)	0.59 (0.49-0.68)
Secondary analysis							
HR-B/V test	86.4 (79.6-92.5)	71.9 (67.7-75.9)	41.9 (38.1-45.8)	95.7 (93.4-97.3)	3.08 (2.63-3.60)	0.19 (0.12-0.30)	0.85 (0.81-0.89)
Procalcitonin measurement	31.6 (23.3-39.5)	87.3 (84.5-90.1)	37.0 (29.2-45.5)	84.5 (82.8-86.1)	2.50 (1.76-3.56)	0.78 (0.69-0.89)	0.62 (0.56-0.67)

Abbreviations: AUROC, area under the receiver operating characteristic curve; HR-B/V, host response bacterial/viral; LR^−^, likelihood ratio negative; LR^+^, likelihood ratio positive; NPV, negative predictive value; PPV, positive predictive value.

Dichotomizing results as either bacterial or viral with a single cutoff value (probability score ≥0.41) would not have accounted for the diagnostic confidence afforded by results in the highest or lowest groups. We therefore analyzed results using the 4-tier likelihood method. A total of 26 bacterial infections (53.1%) were classified as bacterial very likely (probability of >0.73), and 172 viral infections (60.4%) were classified as viral very likely (probability of <0.19) (Table 3; Figure 2B). In the viral very likely group, the NPV for bacterial infection was 98.9% (95% CI, 96.1%-100%). In the bacterial very likely group, the PPV for bacterial infection was 63.4% (95% CI, 47.2%-77.9%) owing to the high prevalence of viral infection (85.3%).

**Table 3.** Performance Characteristics of HR-B/V Test vs Procalcitonin Measurement for the Diagnosis of Bacterial Infection Using a 4-Group Likelihood Scheme

Test and group	No./total No. (%)		% (95% CI)				Interval likelihood ratio (95% CI)^c^
	Bacterial infection	Viral infection	PPV in group^a^	NPV in group^a^	Sensitivity^b^	Specificity^b^	
**Primary analysis**							
HR-B/V test							
VVL (probability score <0.19)	2/49 (4.1)	172/285 (60.4)	NA	98.9 (96.1-100)	NA	60.4 (54.7-66.0)	0.07 (0-0.20)
VL (probability score 0.19-0.40)	3/49 (6.1)	62/285 (21.8)	NA	95.4 (88.1-98.7)	NA	21.8 (17.0-26.9)	0.28 (0-0.77)
BL (probability score 0.41-0.73)	18/49 (36.7)	36/285 (12.6)	33.3 (22.9-48.3)	NA	36.7 (23.4-50.8)	NA	2.91 (1.76-4.74)
BVL (probability score >0.73)	26/49 (53.1)	15/285 (5.3)	63.4 (47.2-77.9)	NA	53.1 (38.8-67.4)	NA	10.08 (5.69-17.36)
Procalcitonin measurement							
BVU (<0.10 ng/mL)	22/49 (44.9)	187/285 (65.6)	NA	89.5 (84.6-93.1)	NA	65.6 (59.9-70.6)	0.68 (0.49-0.94)
BU (0.10-0.24 ng/mL)	13/49 (26.5)	61/285 (21.4)	NA	82.4 (72.0-90.4)	NA	21.4 (17.1-26.8)	1.24 (0.72-2.12)
BL (0.25-0.50 ng/mL)	4/49 (8.2)	13/285 (4.6)	23.5 (6.7-50.0)	NA	8.2 (2.3-18.3)	NA	1.79 (0.41-5.82)
BVL (>0.50 ng/mL)	10/49 (20.4)	24/285 (8.4)	29.4 (15.9-45.6)	NA	20.4 (10.3-33.9)	NA	2.42 (1.17-4.90)
**Secondary analysis**							
HR-B/V test							
VVL (probability score <0.19)	8/117 (6.8)	238/499 (47.7)	NA	96.7 (94.2-98.4)	NA	47.7 (43.6-52.3)	0.14 (0.07-0.27)
VL (probability score 0.19-0.40)	8/117 (6.8)	121/499 (24.2)	NA	93.8 (87.9-96.9)	NA	24.2 (20.7-28.5)	0.28 (0.12-0.56)
BL (probability score 0.41-0.73)	36/117 (30.8)	95/499 (19.0)	27.5 (20.0-35.7)	NA	30.8 (22.6-39.7)	NA	1.62 (1.19-2.27)
BVL (probability score >0.73)	65/117 (55.6)	45/499 (9.0)	59.1 (49.7-67.9)	NA	55.6 (46.5-63.9)	NA	6.16 (4.55-8.59)
Procalcitonin measurement							
BVU (<0.10 ng/mL)	53/117 (45.3)	332/499 (66.5)	NA	86.2 (82.7-89.5)	NA	66.5 (61.9-70.8)	0.68 (0.54-0.82)
BU (0.10-0.24 ng/mL)	27/117 (23.1)	104/499 (20.8)	NA	79.4 (71.7-85.6)	NA	20.8 (17.7-24.7)	1.11 (0.74-1.59)
BL (0.25-0.50 ng/mL)	11/117 (9.4)	30/499 (6.0)	26.8 (15.0-42.8)	NA	9.4 (5.1-15.9)	NA	1.56 (0.75-3.02)
BVL (>0.50 ng/mL)	26/117 (22.2)	33/499 (6.6)	44.1 (30.4-57.1)	NA	22.2 (15.4-30.5)	NA	3.36 (2.06-5.24)

Abbreviations: BL, bacterial likely; BU, bacterial unlikely; BVL, bacterial very likely; BVU, bacterial very unlikely; HR-B/V, host response bacterial/viral; NA, not applicable; NPV, negative predictive value; PPV, positive predictive value; VL, viral likely; VVL, viral very likely.

^a^ PPV and NPV were used for the diagnosis of bacterial infection.

^b^ Sensitivity and specificity were calculated as the percentage of adjudicated participants in each band. The test’s overall sensitivity and specificity were calculated as the sum of values in the respective cells. For example, the HR-B/V test had a sensitivity of 89.8% (53.1% plus 36.7%) for results in the BVL or BL groups and a specificity of 82.2% (60.4% plus 21.8%) for results in the VVL or VL groups.

^c^ Likelihood ratios were used for the diagnosis of bacterial infection. Values in the BVL and BL groups correspond to positive likelihood ratios, whereas values in the VL and VVL correspond to negative likelihood ratios.

Procalcitonin algorithms were also applied to stratify results into 4 likelihood categories (Figure 2A). The cohort was heavily skewed to the groups with low levels of procalcitonin: only 14 participants (28.6%) with bacterial infection were categorized in either of the 2 bacterial diagnostic groups (4 individuals in the bacteria likely group and 10 individuals in the bacteria very likely group) (Table 3; Figure 2C). For participants with procalcitonin levels lower than 0.10 ng/mL (bacterial very unlikely group), the NPV for bacterial infection was 89.5% (95% CI, 84.6%-93.1%). For participants with procalcitonin levels higher than 0.50 ng/mL (bacterial very likely group), we observed a PPV of 29.4% (95% CI, 15.9%-45.6%) for bacterial infection. These values were lower than those observed for the HR-B/V test (*P* < .001 for both comparisons).

#### Secondary Analysis

We next measured test performance among all 616 participants (499 with viral infection and 117 with bacterial infection), including those with a low-confidence adjudication (those without confirmatory microbiological test results or with discordant adjudications) for whom the reference standard was expected to be less accurate. In the full cohort, the HR-B/V test had sensitivity of 86.4% (95% CI, 79.6%-92.5%) and specificity of 71.9% (95% CI, 67.7%-75.9%) when applying a single threshold to discriminate bacterial from viral infection (Figure 2D; Table 2). As observed in the primary analysis, procalcitonin measurement was less sensitive (31.6%; 95% CI, 23.3%-39.5%; *P* < .001) but more specific (87.3%; 95% CI, 84.5%-90.1%; *P* = .005) than the HR-B/V test (Figure 2E), with an NPV of 84.5% (95% CI, 82.8%-86.1%) for bacterial infection. The procalcitonin area under the receiver operating characteristic curve was 0.62 (95% CI, 0.56-0.67) compared with 0.85 (95% CI, 0.81-0.89) for the HR-B/V test (*P* < .001) (eFigure in Supplement 1). When considering all 4 HR-B/V likelihood groups, the viral very likely group had an NPV of 96.7% (95% CI, 94.2%-98.4%), whereas the bacterial very likely group had a PPV of 59.1% (95% CI, 49.7%-67.9%) for bacterial infection. Additional test characteristics are shown in Table 3. A procalcitonin level of lower than 0.10 ng/mL had an NPV of 86.2% (95% CI, 82.7%-89.5%) for bacterial infection (*P* < .001 vs the HR-B/V test). A procalcitonin level higher than 0.50 ng/mL had a PPV of 44.1% (95% CI, 30.4%-57.1%) for bacterial infection (*P* = .005 vs the HR-B/V test).

### Test Performance in Clinical Subgroups

We did not observe any significant differences in HR-B/V test performance based on age, enrollment site, race, ethnicity, or sex in either the primary or secondary analysis (eTable 2 in Supplement 1). Performance was unchanged by the presence of comorbidities, including coronary artery disease, heart failure, chronic kidney disease, chronic lung disease, diabetes, or the composite Charlson Comorbidity Index score. Using hospitalization as a surrogate for illness severity, we observed a higher overall accuracy among hospitalized vs nonhospitalized participants (78.2% vs 73.2%; *P* = .002). Treatment vs nontreatment with antibacterial therapy 8 hours or more before sample collection did not impact test performance as measured by overall accuracy (75.9% vs 74.5%; *P* = .73). Duration of illness, assessed in daily increments, also did not impact test performance (eg, 0-1 days vs 7 days since symptom onset: 76.6% vs 77.3%; *P* = .55). The test was less accurate among participants with enterovirus or rhinovirus (59.3%; 95% CI, 49.9%-68.3%) compared with participants with all other viral infections (90.2%; 95% CI, 85.9%-93.6%; *P* < .001) (eTable 3 in Supplement 1).

### Antibacterial Therapy Use

This study did not directly measure the impact of HR-B/V testing on antibacterial therapy use because testing was not performed in real time. We estimated the potential association if results had been available. In the primary analysis involving 334 participants, 234 individuals were adjudicated as having viral infections, 84 of whom (35.9%) were prescribed antibacterial therapy. These 84 participants represented opportunities for reduced antibacterial medication use. In contrast, the HR-B/V test misclassified 51 participants as having bacterial infections, 30 of whom (58.8%) were not prescribed antibacterial therapy. Therefore, adherence to HR-B/V test results would have had the net result of eliminating inappropriate antibacterial therapy use in 54 of 334 cases (16.2%).

### Classification of COVID-19 Cases

Among 33 participants with acute COVID-19, the HR-B/V test correctly classified 30 participants (90.9%) as having a viral infection (24 in the viral very likely group and 6 in the viral likely group). There was no apparent association between HR-B/V test results and participant-reported symptom severity (Figure 2F). In particular, the symptom severities were similar among the 3 misclassified participants compared with those who were correctly classified as having a viral infection.

## Discussion

This diagnostic study found an NPV of up to 97.9% for bacterial infection using a rapid host gene expression test to discriminate bacterial from viral infection among a large multicenter heterogeneous pediatric and adult population. Antimicrobial resistance is currently increasing at a concerning rate, owing in part to inappropriate use of antibacterial therapy.^6^ Moreover, antibacterial medications pose risks to the individual patient, including allergic reactions, drug-drug interactions, and *Clostridium difficile* infections.^25,26^ To address this diagnostic challenge, we developed a test that discriminated bacterial from viral infection by measuring the host’s gene expression response. This study validated the performance of a research-use-only HR-B/V test for the discrimination of bacterial vs viral infection among 616 participants with febrile ARI. The gene expression test was superior to measurement of procalcitonin, a widely used host response peptide biomarker that has been validated among patients with lower respiratory tract infections. The test also worked equally well among participants with acute COVID-19. Although we did not assess the combined performance of the HR-B/V test and procalcitonin measurement, a previous study^27^ found no improvement vs HR-B/V testing alone.

The potential utility of a test distinguishing bacterial from viral infection is highly dependent on the prevalence of these conditions.^18^ When the prevalence of viral infection is high, as in most cases of ARI, a moderately accurate test would have a high NPV for bacterial infection. As a consequence, such a test does not need to have perfect accuracy to be clinically valuable. For example, despite 55% sensitivity and 76% specificity for procalcitonin measurement to detect bacterial pneumonia, the biomarker’s use has been reported to decrease unnecessary prescription of antibacterial therapy.^13,15^ However, this antibacterial-sparing outcome was not observed in the Procalcitonin Antibiotic Consensus Trial,^14^ a randomized clinical trial of procalcitonin measurement compared with standard of care among patients in the emergency department with lower respiratory tract infections. The reasons for this finding are likely multifactorial, although an insufficient NPV is a likely factor. Compared with procalcitonin measurement, the significantly higher NPV for the HR-B/V test (97.9%) suggests it may have even greater clinical utility, which will need to be verified in a prospective clinical utility study. The most helpful use of the test would therefore be a result indicating the absence of bacterial infection, especially in settings in which the prevalence of such infections is low, as it is with ARI cases. In the present study, we observed a potential reduction of at least 16.2%, even after accounting for false bacterial infection diagnoses in which antibacterial therapy might have been overprescribed. This reduction did not account for scenarios in which clinicians may have chosen to nevertheless prescribe antibacterial therapy, resulting in a lower effect size than we found possible. The test’s potential value will also depend on how easily it can be integrated into clinical workflows.

High rates of antibacterial medication overuse have occurred during the COVID-19 pandemic because of uncertainty about the presence of bacterial infection.^6,7^ Although the HR-B/V test was developed before the pandemic, participants with COVID-19 were identified as having viral infection without a concurrent bacterial infection, offering a tool to support antimicrobial stewardship. We were unable to identify cases of SARS-CoV-2 and bacterial coinfection; therefore, we could not draw any conclusions about test performance in this scenario, although this is an important clinical group to investigate. This caveat applies to cases of bacterial and viral coinfection in general, which were inadequately represented in the present cohort. However, a previous study^16^ found that the HR-B/V test signature identified bacterial infection in 100% of microbiologically confirmed bacterial and viral coinfection. It has also previously been reported that host gene expression can distinguish bacterial, viral, and noninfectious illness in patients with immunocompromising conditions.^28^

One challenge when evaluating tests for bacterial vs viral discrimination is the absence of a diagnostic criterion standard. Adjudication has often been used, with 1 study^29^ reporting reasonable interobserver agreement (κ = 0.88). However, the reliability of this reference decreases in several situations. For example, the absence of microbiological confirmation or discordant adjudications has been associated with lower test accuracy for other biomarkers.^30^ The present study revealed similar findings in which test accuracy decreased among participants with a low-confidence adjudication, defined as having no supportive microbiological results or discordant adjudications (secondary analysis cohort). This decrease was not likely to be associated with inadequate test performance but rather with errors in the reference standard used.

The development and validation of this research-use-only HR-B/V test measured using the BioFire Diagnostics system has been previously described.^16^ The current study confirms those findings while encompassing a broader age range, multiple sites, and a more racially and ethnically heterogeneous cohort. We did not observe any significant differences owing to sex, age, race, ethnicity, enrollment site, comorbidity, or hospitalization status. A previous study^18^ evaluating this test included patients with noninfectious illness. Although inclusion of these patients increased the population available for testing, it came at the expense of test accuracy. In particular, test performance decreased as the number of clinical categories increased.^18,31^ Therefore, the present study focused on the narrower indication of acute (≤7 days) febrile respiratory illness suspected to be caused by bacterial or viral infection.

Measuring gene expression simply, rapidly, and reliably requires the convergence of multiple technologies, including blood cell lysis, RNA purification, RNA preservation or rapid processing, and quantitative real-time reverse transcription amplification (emerging technologies could substitute for some of these elements). This process has been conducted using slower high-complexity research tools, such as low-density array cards (TaqMan; Thermo Fisher Scientific) or nanostring arrays (NanoString Technologies).^28,32,33,34^ There has been more limited progress in the development of rapid sample-to-answer tests. Sutherland et al^17^ described a host gene expression test for the diagnosis of tuberculosis using a molecular analyzer (GeneXpert System; Cepheid). Other studies have described the successful translation of host gene expression tests for viral infection and bacterial vs viral discrimination using 2 different platforms (Qvella Corporation and BioFire Diagnostics, respectively).^16,35^ This previous work included participants with bacterial and viral coinfection, finding the ability to detect host responses to both pathogens. The present study extended those findings, highlighting the opportunity to develop host gene expression–based diagnostic assessments for a variety of clinical applications. Signatures have been described for infectious disease applications (eg, sepsis, candidemia, Dengue, tuberculosis, and malaria)^36,37,38,39,40,41,42,43,44,45,46,47^ and noncommunicable diseases (eg, rheumatic diseases, coronary artery disease, radiation exposure, and cancer).^48,49,50,51,52,53^ As the path from signature discovery to test development and commercialization comes into focus, we expect the development and use of point-of-need host response–based diagnostic tools to expand.

### Limitations

This study has several limitations. These limitations include the use of clinical adjudication as an imperfect reference standard. This study did not address elements of analytical validation, such as site-to-site or run-to-run variability, nor did it evaluate patients with nonrespiratory infections. Furthermore, tests that discriminate bacterial from viral infection are only useful if clinicians modify their antibacterial medication use accordingly. Although we found a reduction in the use of antibacterial therapy is possible, this study did not address clinical utility. We did not identify demographic or clinical variables associated with test performance. Although hospitalization as a marker of severity did not impact test performance, there were too few participants with critical illness defined by intensive care unit admission to evaluate the test in this subgroup. There were also too few participants with bacterial and viral coinfection to compare results with those of other published reports. Serial HR-B/V test measurements would help to define a role for the test in treatment-response monitoring.

## Conclusions

This diagnostic study found that host gene expression could be measured at the point of need using a readily available clinical platform. The gene expression biomarkers included in the HR-B/V test accurately distinguished bacterial from viral infections among participants with acute febrile respiratory tract infections. Test performance was superior to procalcitonin measurement and was also accurate among participants with COVID-19.

## Notes

Supplement 1. **eMethods.** Study Design and Reference Standard **eTable 1.** Distribution of Participants in Primary and Secondary Analyses by Adjudication Confidence and Microbiological Cause **eTable 2.** HR-B/V Test Performance Stratified by Demographic or Clinical Parameters **eTable 3****.** Microbiological Causes and Associated HR-B/V Test Accuracies **eFigure.** Area Under the Receiver Operating Characteristic Curves for HR-B/V Test and Procalcitonin Measurement in the Primary Analysis and Secondary Analysis Cohorts

Supplement 2.
Nonauthor Collaborators
