# Supplementary material for: Prospective Validation of a Rapid Host Gene Expression Test to Discriminate Bacterial From Viral Respiratory Infection
Source: JAMA Netw Open. 2022 Apr 14;5(4):e227299. doi: 10.1001/jamanetworkopen.2022.7299 (PMC9011121; doi:10.1001/jamanetworkopen.2022.7299)
Supplement: Supplement 1. — eMethods. Study Design and Reference Standard eTable 1. Distribution of Participants in Primary and Secondary Analyses by Adjudication Confidence and Microbiological Cause eTable 2. HR-B/V Test Performance Stratified by Demographic or Clinical Parameters eTable 3. Microbiological Causes and Associated HR-B/V Test Accuracies eFigure. Area Under the Receiver Operating Characteristic Curves for HR-B/V Test and Procalcitonin Measurement in the Primary Analysis and Secondary Analysis Cohorts [file jamanetwopen-e227299-s001.pdf]

Supplementary Online Content

Ko ER, Henao R, Frankey K, et al; Antibacterial Resistance Leadership Group. Prospective validation of a rapid host gene expression test to discriminate bacterial from viral respiratory infection. *JAMA Netw Open*. 2022;5(4):e227299. doi:10.1001/jamanetworkopen.2022.7299

eMethods. Study Design and Reference Standard

eTable 1. Distribution of Participants in Primary and Secondary Analyses by Adjudication Confidence and Microbiological Cause

eTable 2. HR-B/V Test Performance Stratified by Demographic or Clinical Parameters

eTable 3. Microbiological Causes and Associated HR-B/V Test Accuracies

eFigure. Area Under the Receiver Operating Characteristic Curves for HR-B/V Test and Procalcitonin Measurement in the Primary Analysis and Secondary Analysis Cohorts

This supplementary material has been provided by the authors to give readers additional information about their work.

© 2022 Ko ER et al. *JAMA Network Open*.

eMethods. Study Design and Reference Standard

Study Design

Subjects were enrolled from 2014-2019 at Duke University Hospital and the Durham VA Health Care System (Durham, NC), and at eight additional emergency departments from 2017-2019: UNC Health Care (Chapel Hill, NC), Henry Ford Health System (Detroit, MI), Brigham and Women's Hospital (Boston, MA), The University of California Hospital at Davis (Sacramento, CA), Children's Memorial Hermann (McGovern Medical) (Houston, TX), Hasbro Children's Hospital (Providence, RI), The University of Utah Medical Center (Salt Lake City, UT), Children's Hospital of Pittsburgh (Pittsburgh, PA), and Newton-Wellesley Hospital (Newton, MA). Subjects were enrolled as part of RADICAL-2 (Rapid Diagnostics in Categorizing Acute Lung Infection) (ClinicalTrials.gov NCT03192072), which had broader inclusion criteria such as duration of illness up to 28 days, did not require the presence of fever, and allowed for participants with non-infectious illness. Sample sizes were calculated for the larger RADICAL-2 cohort to estimate HR-B/V test performance and therefore do not apply to the subset described here.

Qualifying symptoms included headache, rhinorrhea, nasal congestion, sneezing, sore throat, itchy/watery eyes, conjunctivitis, cough, shortness of breath, sputum production, chest pain, and wheezing. Qualifying vital sign abnormalities included heart rate ≥90 (or ≥110 for children aged 2-6 years), respiratory rate ≥20, and temperature ≥38.0ºC or ≤36ºC.

Reference Standard

Adjudicators were experts in infectious disease, pulmonary/critical care, hospital medicine, or emergency medicine. Adjudication of subjects <18 years included ≥1 reviewer with pediatric expertise. Adjudicators had access to the full medical record. Results of study-specific supplemental testing included the BinaxNOW S. pneumoniae urinary antigen test (Alere, Waltham, MA) and a multiplex respiratory pathogen panel (Respiratory Pathogen Panel, Luminex, Austin, TX) performed on a nasopharyngeal swab. Adjudications were performed ≥28 days after enrollment. Procalcitonin was measured in all participants using the miniVIDAS immunoanalyzer (bioMérieux, Marcy-l'Étoile, France) but was not provided to adjudicators. However, in 24 (3.9%) cases, procalcitonin was measured clinically and would have been available during adjudication. HR-B/V test results were not provided to adjudicators. Adjudicator discordance was resolved by a consensus panel of at least three experts. Cases were considered indeterminate when adjudicators could not determine the underlying etiology.

© 2022 Ko ER et al. *JAMA Network Open*.

eTable 1. Distribution of Participants in Primary and Secondary Analyses by Adjudication Confidence and Microbiological Cause

Adjudication Confidence[a]	Bacterial		Viral	
	Microbiology positive	Microbiology negative	Microbiology positive	Microbiology negative
Primary Analysis (n=334)				
High Confidence Adjudication (n=334)	49	n/a[b]	285	n/a[b]
Secondary Analysis (n=616)				
High Confidence Adjudication (n=334)	49	n/a[b]	285	n/a[b]
Low Confidence Adjudication (n=282)	8	60	57	157

[a] High confidence adjudication includes subjects with concordant adjudications and an identified microbiological etiology. Low confidence adjudications include subjects with discordant adjudications or no identified microbiological etiology.

[b] Not applicable since subjects with a high confidence adjudication were required to have an identified microbiological etiology.

© 2022 Ko ER et al. *JAMA Network Open*.

eTable 2. HR-B/V Test Performance Stratified by Demographic or Clinical Parameters

Parameter (n)[a]	Accuracy (%)[b]	P-value[c]
Age		0.95
<12 years (135)	79.3	
12-20 years (130)	73.1	
21-64 years (301)	73.4	
≥65 years (50)	74.0	
Gender		0.10
Female (292)	76.0	
Male (324)	73.5	
Race		0.53
White (245)	71.8	
Black (293)	76.8	
American Indian/Alaska Native (11)	72.7	
Asian (11)	81.8	
Ethnicity		0.50
Non-Hispanic (502)	74.3	
Hispanic (110)	76.4	
Site		0.61
Duke University (223)	73.1	
Durham VA Health Care System (97)	66.0	
Henry Ford Hospital (72)	80.6	
UNC Chapel Hill (1)	100	
Brigham & Women's Hospital (60)	81.7	
University of Utah (2)	100	
Children's Memorial Hermann (76)	76.3	
Newton Wellesley (3)	100	
University of California, Davis (19)	89.5	
Hasbro Children's Hospital (47)	68.1	
Children's Hospital of Pittsburgh (16)	81.3	
Comorbidities		
Coronary Artery Disease (35)	85.7	0.40
Congestive Heart Failure (27)	81.5	0.94
Chronic Kidney Disease (25)	80.0	0.97
Chronic Lung Disease (156)	69.9	0.18
Diabetes Mellitus (62)	77.4	0.46
Charlson Comorbidity Index >0 (289)	73.4	0.88
Hospitalization		0.002
No (433)	73.2	
Yes (179)	78.2	
Prior Antibacterial Therapy[d]		0.73
Received (87)	75.9	
Not received (529)	74.5	
Days Since Symptom Onset		0.55
0-1 (141)	76.6	
2 (140)	74.3	
3 (129)	73.6	
4 (73)	74.0	
5 (44)	72.7	
6 (21)	66.7	
7 (66)	77.3	

© 2022 Ko ER et al. *JAMA Network Open*.

[a] All values are based on the 616-subject study cohort, which corresponds to the secondary analysis cohort. Numbers within each category may not sum to 616 due to missing data.

[b] Accuracy was calculated based on the number of bacterial or viral infections classified correctly using a single-threshold scheme where the Bacterial Very Likely and Bacterial Likely groups comprised a Bacterial diagnosis and the Viral Very Likely and Viral Likely groups comprised a Viral diagnosis.

[c] P-values for age, gender, race, ethnicity, site, comorbidities, hospitalization, and days since symptom onset were calculated using ANOVA.

[d] Prior antibacterial therapy is defined as any systemically active antibacterial taken by or administered to the patient at least eight hours before sample collection for HR-B/V testing. P-value was calculated using ANOVA comparing the accuracies in those who did or did not receive prior antibacterial therapy.

© 2022 Ko ER et al. *JAMA Network Open*.

eTable 3. Microbiological Causes and Associated HR-B/V Test Accuracies

**Bacterial Etiologies** (n=60)[a]	**Number of cases**	**HR-B/V Accuracy (%)**[b]
Streptococcus pyogenes	26	24/26 (92.3)
Streptococcus, other	8	7/8 (87.5)
Polymicrobial	6	5/6 (83.3)
Escherichia coli	5	4/5 (80)
Mycoplasma pneumoniae	4	2/4 (50)
Staphylococcus aureus	4	4/4 (100)
Enterococcus faecalis	1	1/1 (100)
Fusobacterium necrophorum	1	0/1 (0)
Haemophilus influenza	1	1/1 (100)
Legionella sp.	1	1/1 (100)
Proteus mirabilis	1	1/1 (100)
Pseudomonas aeruginosa	1	1/1 (100)
Salmonella sp.	1	1/1 (100)
**Viral Etiologies** (n=373)[a]	**Number of cases**	
Influenza	120	110/120 (91.7)
Enterovirus/rhinovirus	118	70/118 (59.3)
SARS-CoV-2	33	30/33 (90.9)
Metapneumovirus	31	27/31 (87.1)
Coronavirus (non-SARS-CoV-2)	19	17/19 (89.5)
Respiratory Syncytial Virus	17	16/17 (94.1)
Adenovirus	13	12/13 (92.3)
Parainfluenza	10	9/10 (90)
Epstein Barr Virus	8	6/8 (75)
Bocavirus	3	2/3 (66.7)
Cytomegalovirus	1	1/1 (100)

[a] Etiologies are based on results of urinary streptococcal antigen testing, respiratory pathogen panel testing, and routine clinical care.

[b] Accuracy is defined as the number of subjects assigned to the correct etiologic class out of all subjects with the specified pathogen.

© 2022 Ko ER et al. *JAMA Network Open*.

eFigure. Area Under the Receiver Operating Characteristic Curves for HR-B/V Test and Procalcitonin Measurement in the Primary Analysis (A) and Secondary Analysis (B) Cohorts

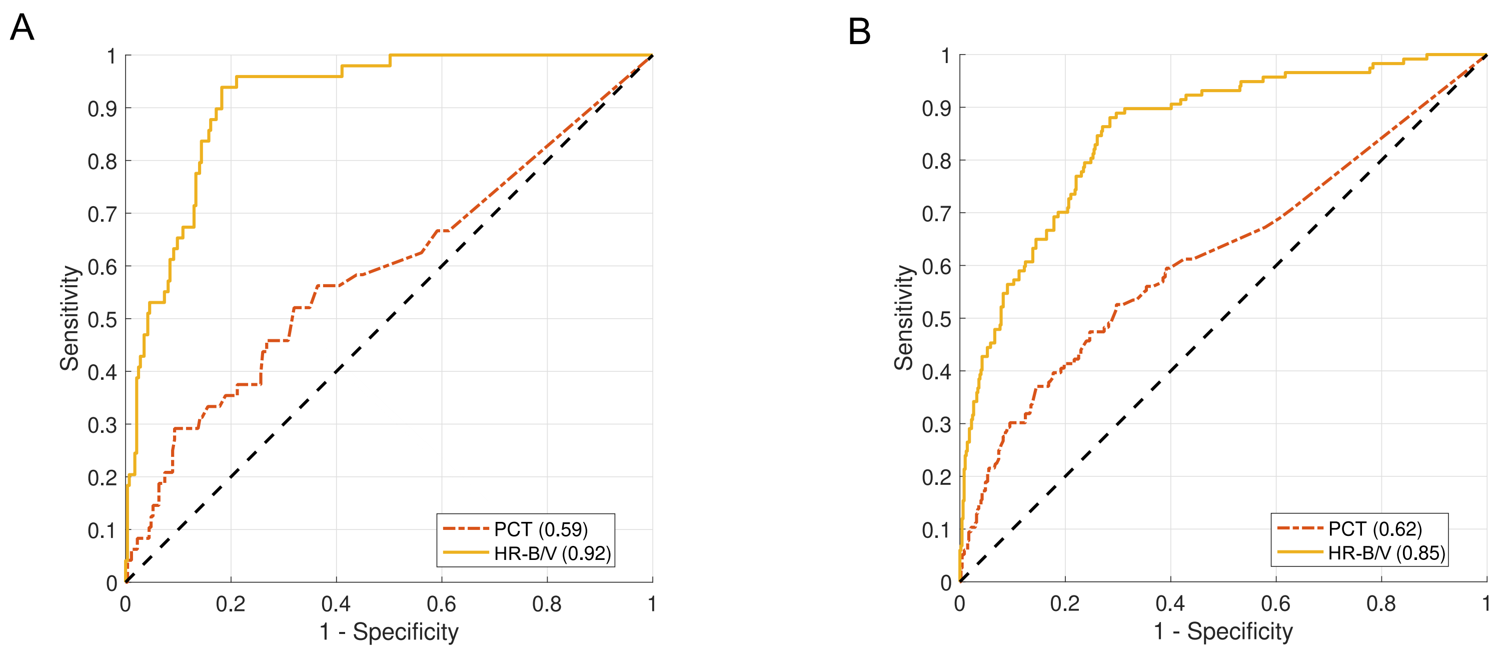

© 2022 Ko ER et al. *JAMA Network Open*.
