# Supplementary material for: Prospective Validation of a Rapid Host Gene Expression Test to Discriminate Bacterial From Viral Respiratory Infection
Source: JAMA Netw Open. 2022 Apr 14;5(4):e227299. doi: 10.1001/jamanetworkopen.2022.7299 (PMC9011121; doi:10.1001/jamanetworkopen.2022.7299)
Supplement: Supplement 2. — Nonauthor Collaborators [file jamanetwopen-e227299-s002.pdf]

Supplemental Online Content: Nonauthor Collaborators

*Indicates required information. Only first name, last name, and suffix will appear in PubMed.

*Group Name(s): Antibacterial Resistance Leadership Group							
***First Name and Middle Initial(s)**	***Last Name**	***Suffix (eg, Jr, III)**	Academic Degrees	Institution	Location (city, state/province, country)	Role or Contribution, eg, chair, principal investigator	Group (if more than 1 Group listed in the byline) and/or Subgroup (eg, Steering Committee)
Henry F	Chambers	III	MD	University of California San Francisco			
Scott R	Evans		PhD	George Washington University			
Vance G	Fowler	Jr	MD, MHS	Duke University			
Toshimitsu	Hamasaki		PhD	George Washington University			
Robin	Robin		MD	Mayo Clinic			
Heather R	Cross		DPhil	Duke University			
Anthony D	Harris		MD, MPH	University of Maryland			
Melinda M	Pettigrew		PhD	Yale School of Public Health			
David	van Duin		MD, PhD	University of North Carolina, Chapel Hill			
Helen W	Boucher		MD	Tufts University			
Dennis M	Dixon		PhD	NIH			
Clayton C	Huntley		PhD	NIH			
Varduhi	Ghazaryan		MD, MPH	NIH			
Lanling	Zou		MD, PhD	NIH			
Erica L	Raterman		PhD	NIH			
Tamika K	Samuel		PhD	NIH			
Kimberly E	Hanson		MD, MHS	University of Utah			
Yohei	Doi		MD, PhD	University of Pittsburgh			
Loren G	Miller		MD, MPH	University of California, Los Angeles			
Tom P	Lodise		PharmD, PhD	Albany College of Pharmacy and Health Sciences			
Samuel A	Shelburne		MD, PhD	MD Anderson Cancer Center			
Ritu	Banarjee		MD, PhD	Vanderbilt University			
Sara E	Cosgrove		MD, MS	Johns Hopkins School of Medicine			
David L	Paterson		MBBS, PhD	University of Queensland			
Ebbing	Lautenbach		MD, MPH, MSCE	Perelman School of Medicine			
